# Influence of a Hydroactive Lipogel on Color Distribution and Color Intensity of Tattoo Pigments: Results From an Exploratory Ex Vivo Approach

**DOI:** 10.1111/jocd.70406

**Published:** 2025-08-22

**Authors:** Lisa Kümper, Nadine Roese, Karin Pappelbaum, Janin Edelkamp, Marta Bertolini

**Affiliations:** ^1^ MEDICE Arzneimittel Pütter GmbH & Co. KG Iserlohn Germany; ^2^ QIMA Life Sciences, QIMA Monasterium GmbH Münster Germany

To the Editor,

After tattooing, adequate hygiene and wound management are critical for wound healing and the establishment of a satisfying aesthetic appearance of the tattoo. The European standards on safe and hygienic practice for tattooing recommend moist wound healing using a hypoallergenic ointment or a non‐perfumed lotion [1]. However, most creams or ointments available for moist wound healing have not been tested for their influence on the appearance of freshly engraved tattoos.

The topical hydroactive lipogel “MediGel Wund‐ und Heilgel” supports moisturization and acidification of the wound environment and therewith epithelialization of the wound [2]. Its beneficial effects on wound healing have been proven in controlled studies and real‐life models of incisive wounds [3, 4]. As a certified medical device, it is indicated for the treatment of acute wounds such as abrasions and cuts as well as first and second degree burns.

In this exploratory study, we used an ex vivo study approach to investigate any influence of our hydroactive lipogel on ink distribution (relevant for blurred lines) and tattoo pigment intensity (relevant for color changes) in freshly tattooed skin.

Human, abdominal skin samples were obtained from one Caucasian female (45 years old) after informed written consent and ethics committee approval. Skin was challenged with a dermaroller to mimic typical tattoo wounds before taking skin punches. Black, blue, or red diluted tattoo ink was injected into each punch twice at day 0. Freshly tattooed skin either remained untreated or lipogel was topically applied. Afterwards, skin samples were frozen or organ cultured for 3, 12, and 48 h [5]. Cryosections were used for histopathological examination of skin integrity (hematoxylin/eosin staining, data not shown) or for measurement of ink intensity and spatial diffusion of colored area. Imaging was performed using BZ‐X800, and analysis was performed with ImageJ software (National Institutes of Health, Bethesda, MD, USA) [6].

Representative images showed that ink injection was technically comparable between skin punches (see Figure 1a). Dermaroller challenge did not lead to histologically visible wounding effects, and skin integrity remained unaltered after ink injection and lipogel treatment. The spatial distribution of ink directly upon injection, measured as the distance between the ink‐containing area and epidermis, varied between 294 and 1392 μm (see Figure 1b). However, variations were comparable between untreated and lipogel‐treated skin biopsies, indicating that lipogel application does not affect the localization of tattoo pigments in the dermis.

**FIGURE 1:**
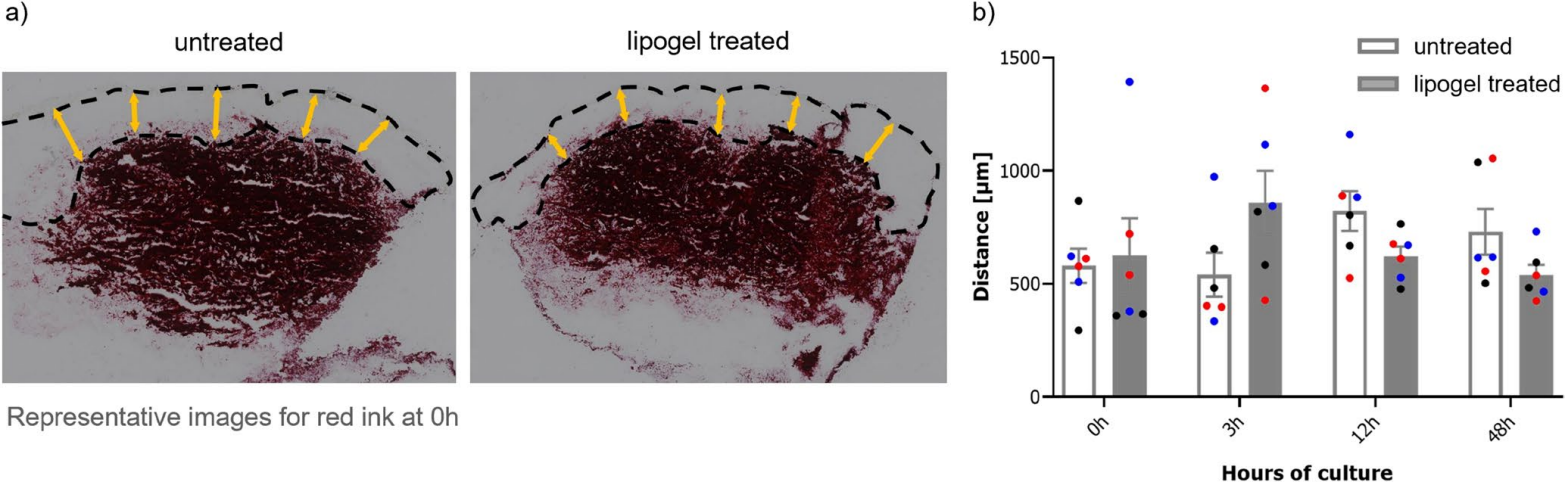
Distance of ink containing area in the dermis to epidermis in biopsies with and without lipogel treatment. (a) Representative images of selected distances to measure color diffusion in the skin (yellow arrows). (b) Averaged distance for all three ink colors (black, blue and red marks) in untreated and lipogel treated skin biopsies at specific timepoints. Black: KURO SUMI black; blue: KURO SUMI blue; red: KURO SUMI red. Data are presented as mean ± SEM.

In untreated punches, the average color intensity increased during organ culture with a strong increment between 0 h and 3 h (see Figure 2b), in line with the known “tattoo settling” effect experienced in subjects after tattooing. Interestingly, such an increase in intensity over time did not occur in punches treated with lipogel as tattoo aftercare: the initial ink intensity in punches treated with lipogel was remarkably higher at 0 h compared to untreated punches, showing a similar level to the 3 h untreated organ cultured samples. Ink intensities were comparable between untreated and lipogel treated biopsies at all later time points analyzed (3, 12, and 48 h). Thus, these preliminary results suggest that lipogel as tattoo aftercare might contribute to color stability.

**FIGURE 2:**
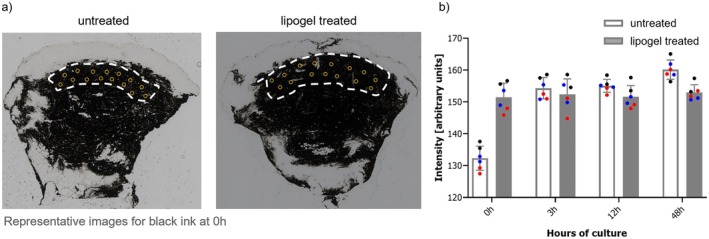
Ink color intensity after ink injection in biopsies with and without lipogel treatment. (a) Representative images of selected measurement points (yellow circles). (b) Averaged intensities for all three ink colors (black, blue and red marks) at specific timepoints. Black: KURO SUMI black; blue: KURO SUMI blue; red: KURO SUMI red. Data are presented as mean ± SEM.

Recognizing the need to repeat experiments with skin samples from more donors and to investigate longer time points, our pilot data demonstrate how the human skin organ culture model can aid in understanding changes occurring in tattooed skin and in testing assets that may influence these processes.

## 1 Conclusion

The results of this pilot ex vivo study indicate no influence of our hydroactive lipogel on tattoo pigment distribution and intensity and therewith the aesthetic appearance of freshly engraved tattoos. As a conclusion, our hydroactive lipogel seems to be suitable for supporting wound healing after tattooing.

## Disclosure

*A Statement of Contribution*: MEDICE Arzneimittel Pütter GmbH & Co. KG funded the study and was involved in project conception, interpretation of data, manuscript drafting, and critical revision. QIMA Life Sciences, QIMA Monasterium GmbH planned and conducted the study as a contract research organization and was involved in data interpretation and critical revision of the manuscript.

## Ethics Statement

Human skin samples have been obtained after informed, written patient consent under ethics committee approval (Medical Association of Westfalen‐Lippe and Westfälischen Wilhelms University Münster; Monasterium Biobank 2019‐297‐f‐S; Study protocol 2018‐483‐f‐S). This study was conducted according to the Declaration of Helsinki principles.

## Conflicts of Interest

L.K. and N.R. are employees of the sponsor. K.P., J.E., and M.B. are employees of QIMA Monasterium GmbH.

## Data Availability

The data that support the findings of this study are available from the corresponding author upon reasonable request.
